# A research program-linked, course-based undergraduate research experience that allows undergraduates to participate in current research on mycobacterial gene regulation

**DOI:** 10.3389/fmicb.2022.1025250

**Published:** 2023-01-06

**Authors:** Louis Anthony Roberts, Scarlet S. Shell

**Affiliations:** ^1^Department of Biology and Biotechnology, Worcester Polytechnic Institute, Worcester, MA, United States; ^2^Bioinformatics and Computational Biology Program, Worcester Polytechnic Institute, Worcester, MA, United States

**Keywords:** authentic research, undergraduate laboratory teaching, assessments, molecular biology, CRISPRi, RNA, gene expression, *Mycolicibacterium smegmatis*

## Abstract

Undergraduate instructional biology laboratories are typically taught within two paradigms. Some labs focus on protocols and techniques delivered in “cookbook” format with defined experimental outcomes. There is increasing momentum to alternatively employ student-driven, open-ended, and discovery-based strategies, often *via* course-based undergraduate research experiences (CUREs) using crowd-sourcing initiatives. A fraction of students also participate in funded research in faculty research labs, where they have opportunities to work on projects designed to expand the frontiers of human knowledge. These experiences are widely recognized as valuable but are not scalable, as most institutions have many more undergraduates than research lab positions. We sought to address this gap through our department’s curriculum by creating an opportunity for students to participate in the real-world research process within a laboratory course. We conceived, developed, and delivered an authentic, guided research experience to students in an upper-level molecular biology laboratory course. We refer to this model as a “research program-linked CURE.” The research questions come directly from a faculty member’s research lab and evolve along with that research program. Students study post-transcriptional regulation in mycobacteria. We use current molecular biology methodologies to test hypotheses like “UTRs affect RNA and protein expression levels,” “there is functional redundancy among RNA helicases,” and “carbon starvation alters mRNA 5′ end chemistries.” We conducted standard assessments and developed a customized “Skills and Concepts Inventory” survey to gauge how well the course met our student learning outcomes. We report the results of our assessments and describe challenges addressed during development and execution of the course, including organizing activities to fit within an instructional lab, balancing breadth with depth, and maintaining authenticity while giving students the experience of obtaining interpretable and novel results. Our data suggest student learning was enhanced through this truly authentic research approach. Further, students were able to perceive they were participants and contributors within an active research paradigm. Students reported increases in their self-identification as scientists, and a positive impact on their career trajectories. An additional benefit was reciprocation back to the funded research laboratory, by funneling course alumni, results, materials, and protocols.

## 1. Introduction

The motivation and rationale for creating a truly authentic research course lies in the national call that originated nearly two decades ago to transform undergraduate science teaching. Among the recommendations included in the President’s Council of Advisors on Science and Technology report Engage to Excel (Gates Jr. and Mirkin, 2012), and in the AAAS/NSF report Vision and Change in Undergraduate Biology Education (Brewer and Smith, 2011), is replacing standard laboratory courses with discovery-based research courses. Toward this goal, our department joined the Small World/Tiny Earth Initiative (Tiny Earth Initiative, 2022) in 2015, and Science Education Alliance- Phage Hunters Advancing Genomics and Evolution (SEA-PHAGES, 2022) in 2016. These crowd-sourcing labs were well-suited for revamping our introductory lab curriculum, allowing our students to explore discovery-based science. These courses were well-received by the students, as evidenced by CURE and self-assessment data [Buckholt et al., 2022 (in press)]. The success of these courses inspired us to thoughtfully create advanced courses adhering to the same principle of discovery.

**Figure 1:**
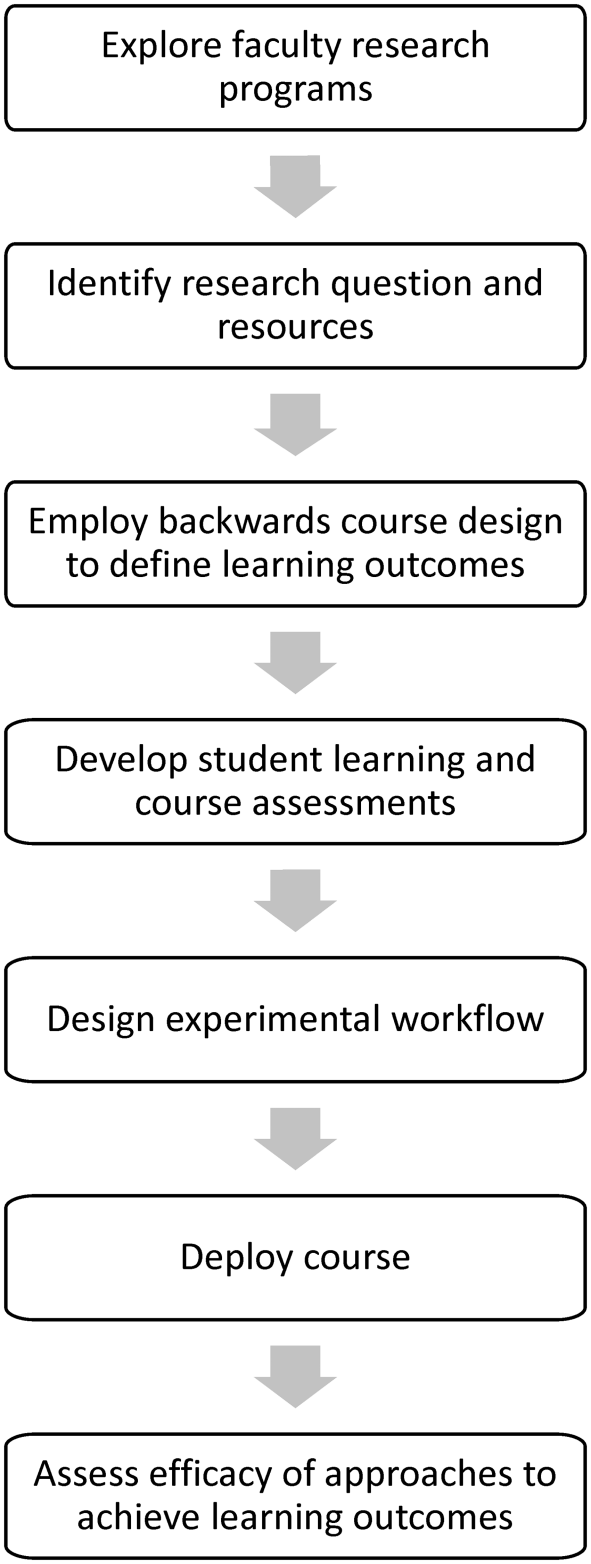
Workflow for creating an rpl-CURE.

A significant challenge in laboratory science education is identifying a mechanism to provide the opportunity for all students to gain an understanding of how life science research is conducted. While a fraction of our majors undertake projects in the laboratories of our research-active faculty, this model is not scalable to accommodate and train all our students authentically. At the upper level we sought to design our own authentic research labs, based on the enterprises of our research-active, extramurally funded faculty. This approach would allow us to have a more focused and applied paradigm, in which students would conduct original research projects contributing to funded research initiatives. This model shares many goals and has a degree of similarity with authentic research experiences such as the Freshman Research Initiative at the University of Texas-Austin (Rodenbusch et al., 2016). In our case we wished to create a new laboratory course that exists independently from a university-sponsored program or course sequence, could be offered with existing departmental resources, and would serve upper-level students majoring in biology and related disciplines. We held a faculty retreat and brainstormed to identify facets of research programs that were intellectually and temporally suitable for, and scalable to, our existing laboratory environment. We emerged with the consensus that we could extract elements of current research from the programs of our faculty and deploy them in a laboratory course setting. We are defining these as research program-linked CUREs (rpl-CUREs). A signature of these courses is that they maintain the authenticity of funded research projects, while giving many more students the experience of obtaining interpretable and novel results within the constraints of a lab course. The data and discoveries the students generate transcend the course, contributing to active research projects. The workflow we followed to create our course is depicted in Figure 1.

Based upon the considerations and constraints detailed herein, and the necessity for a molecular biology wet lab which was absent from our curriculum, we fashioned a new course called “Molecular Biology and Genetic Engineering: Approaches and Applications” (MBGE) based on the research program of Dr. Scarlet Shell, who investigates mRNA-level gene regulation in the tuberculosis model, *Mycolicibacterium smegmatis*. Tuberculosis kills 1.3 million people around the world each year (WHO, 2021) and is challenging to treat with antibiotics due to its microenvironment in the granuloma and slow-growing nature. One avenue to enhance treatment efficacies and options is to learn how the disease agent *Mycobacterium tuberculosis* regulates gene expression in response to stress. The main research question we are addressing is “how do mRNA metabolism and post-transcriptional regulation of gene expression contribute to stress tolerance in mycobacteria?” We use molecular and genetic approaches to address this question from a mechanistic perspective.

After identifying the faculty research program and scientific questions we would address in the course, we used the principles of backwards course design (Davidovitch, 2013) to define learning outcomes (Table 1). We used standard and unique lab course assessments to determine if we met our goals; namely that the students undertake an open-ended project for which they would have ownership, is of high value to others, advances knowledge in the field through new discovery, and is transferrable back into a research setting. We will report on our assessments and follow the extension of the research findings to the laboratory of the research-active faculty member. Given the rapidly evolving nature of funded research, we have offered three unique variants of the course over the past 6 years. Here we describe the commonalities across all versions, as well as elements unique to each thrust. From the perspectives of the instructor, funded faculty member, and the students, our assessments demonstrate the efficacy of this approach.

**Table 1:** Learning outcomes mapped to student assessments.

Upon completion of this course, students should be able to…	How Assessed
1. Demonstrate mastery of the quantitative and procedural skills related to molecular biology	Direct observation by teaching staff; pre-lab quizzes; successful experimental outcomes
2. Design appropriate experiments using contemporary approaches and techniques in molecular biology and genetic engineering	Proper design of primers, oligos, and experiments based upon discussion and lab notebook checks
3. Properly collect, record, and analyze experimental data to assess the validity of a scientific hypothesis	Lab notebooks, weekly “lab meeting” informal presentations, and final poster
4. Present findings clearly in written and verbal formats while adhering to the standards, style, and intellectual honesty expected of life scientists	Daily summaries; weekly “lab meetings” and final poster
5. Function effectively, safely, and collaboratively as part of a team of scientists	Lab notebook and poster presentation
6. Gain exposure to how research directions and chosen and experiments are designed in academic research laboratories	Student comments *via* university course evaluations and personal reflections

## 2. Materials and methods

### 2.1. Molecular biology and genetic engineering course setting, enrollments, and mechanics

The academic year at our institution Worcester Polytechnic Institute (WPI) is divided into quarters of seven class weeks. The MBGE lab was structured such that students attend three 3-h blocks per week, yielding 63 contact hours. Students typically require the full lab period to complete their experiments. Some monitoring of cultures (e.g., for growth curves) occurs outside of scheduled lab periods. The expectation at our institution is that students spend ~15 h per week on each of their three classes. Thus, most assessments of student learning were based on in-lab activities (e.g., notebooks and participation), and succinct pre-lab quizzes and retrospective summaries. A list of these assessments is provided in Table 2.

**Table 2:** Student assessments and relative weights.

Assessment	Occurrence and Group (G) vs. Individual (I)	Weight percent
Prelab quizzes	Bi-weekly (I)	25
Lab notebooks (Benchling)	Weekly (G)	25
Research poster	End of course with iterative feedback steps of outlining, sketching, and drafting (G)	30
Project summaries and personal reflection	Summaries weekly in lab notebook (G); personal reflection at end of course (I)	10
Participation and safety	Direct observation by teaching staff; completion of safety unit and quiz (I)	10

(I) = individual assignment; (G) = group assignment.

Enrollment for the first offering (2017) was capped at 12 students. In subsequent years the capacity was increased, and 17 students completed the course each year. Students were paired, with one group of three as necessitated. Over the course of all six offerings, ~75% of students were biology/biotechnology majors, ~10% were biochemistry majors, and the remaining ~15% were majoring in bioinformatics and computational biology, engineering (i.e., biomedical, chemical, and mechanical), and environmental science.

**Figure 2:**
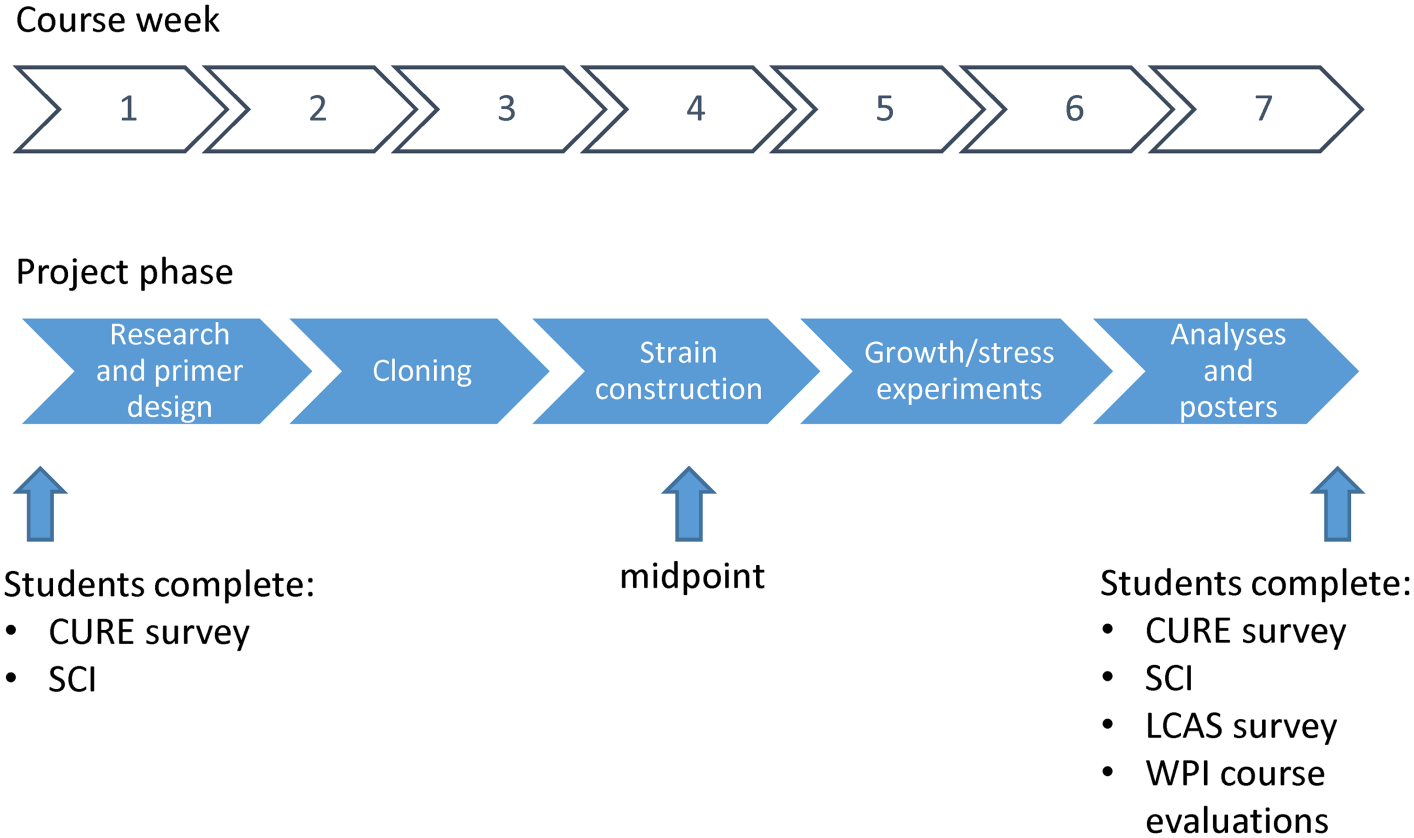
General timeline of student experiments and course assessments.

Generally, the first 3 weeks were devoted to DNA manipulation and strain construction, followed by 3 weeks of analysis of gene expression or growth phenotypes. Each term we focused on no more than two outputs, detailed for each experimental thrust below. The final week was utilized to refine analyses, catalog findings, and design, print, and present student research posters. The timeline for the general student workflow is depicted in Figure 2.

### 2.2. Personnel and space

Dr. Roberts (associate professor of teaching) coordinated, designed, and instructed the MBGE course. Dr. Shell provided the project ideas and experimental approach. The course design process was completed over 8 months. Meetings were held monthly from conception to launch, and instructional and assessment design teams were consulted as appropriate. Dr. Shell attended the first lab day to present the context of the research, attended the culminating poster session, and provided feedback and advice during the course as needed. Shell lab members provided guidance, constructs, and strains. One full-time (15-20 h/wk) graduate student teaching assistant was assigned to the course; in some offerings one undergraduate teaching assistant was hired hourly to assist in the lab and with grading.

The laboratory space (~900 sq. ft) contains 10 student benches, with 20 seats in total. The wet lab is equipped with two thermocyclers, two growth chambers, two shaking incubators, agarose gel electrophoresis and SDS-PAGE units, blue light boxes, two 4°C, one −20°C, and one −80°C. Laptop computers are available to the students. A fluorescent plate reader (Victor3, PerkinElmer) and microscope (Zeiss), each equipped with appropriate filters, an electroporator, and a gel documentation system (BioRad) are available in the same building as the lab class. Dry ice, wet ice, and liquid nitrogen are also available in the building. We utilize the FastPrep-24 5G instrument (MP Biomedical) to extract RNA for qPCR and proteins for SDS-PAGE/immunoblot, and the qPCR thermocycler (Applied Biosystems 7,500) present in the Shell lab. Instructional staff prepare all base media and components.

### 2.3. *Mycolicibacterium smegmatis* growth, genetic manipulation, and resources

*M. smegmatis* is a soil bacterium that is a safe (BSL-1), tractable, and widely used model for *M. tuberculosis* (BSL-2/3). The genome of *M. smegmatis* is contained on a single, circular chromosome approximately 7 Mbp in length. *M. smegmatis* grows rapidly in liquid culture (doubling time of the mc^2^155 strain is ~2.8 h at 37°C vs. ~18 h for *M. tuberculosis*), and colonies appear within 2–3 days of streak plating and within 5 days after transformation. Electroporation of competent *M. smegmatis* is efficient; single copy integration of plasmids into the genome is targeted at the L5 and Giles phage integration sites.

For PCR and DNA assembly, we used 2X versions of Q5, Taq, and HiFi Assembly master mixes from New England Biolabs (NEB). Maximum chemically competent *E. coli* were purchased from NEB (C2987I), while electrocompetent *M. smegmatis* were prepared by the students. Reagents for RNA extraction/purification (2 ml disruption tubes from OPS Diagnostics; 100 μm zirconium lysing matrix, molecular grade) and qPCR reagents (e.g., iTaq SYBR Green from Bio-Rad) were also required. Oligonucleotides were obtained from Integrated DNA Technologies or Eton Biosciences; DNA sequencing reactions (~40 per offering) were performed by Eton Bioscience and Quintarabio. The free web-based platform Benchling (Benchling Biology Software, 2022) was utilized by students for DNA sequence handling, annotation, and alignment. We also used the electronic lab notebook function within Benchling for students to integrate their notes, protocols, images, and analyses in a scientifically acceptable format. Information on genes, transcripts, and nucleotide sequences was obtained from Mycobrowser (Kapopoulou et al., 2011) and PATRIC (Wattam et al., 2014); secondary structure predictions were made using Sfold (Ding et al., 2004).

## 3. Results and discussion

### 3.1. Defining learning outcomes, assessments, and assignments

At WPI we use the principle of “backwards course design” (Davidovitch, 2013), in which learning outcomes are identified first, then the class is built within that framework to accomplish the stated objectives. We identified six learning outcomes (presented in Table 1) for this class. We wrote the learning outcomes and course description to be adaptable to altering the focus, approach, and methodologies contained in the course. This would allow us to utilize different projects or research programs in the future. Our courses do not have prerequisites nor restrictions by major, so sequencing in the curriculum was not a determining factor. Lab courses offered by our department are completely independent from our lecture classes. The lack of restrictions motivates our laboratory instructors to train students with a broad distribution of incoming skills and knowledge and provides many degrees of freedom with respect to course content. We focused on creating an upper-level lab, which selects for students who have completed at least one lower-level labs first. Recommended background includes the lecture classes in molecular biology, genetics, microbiology, and cell biology; the crowd-sourcing Tiny Earth Initiative course and “Enzymes, Proteins, and Purification” (two of our lower-level offerings) are recommended lab experiences.

**Figure 3:**
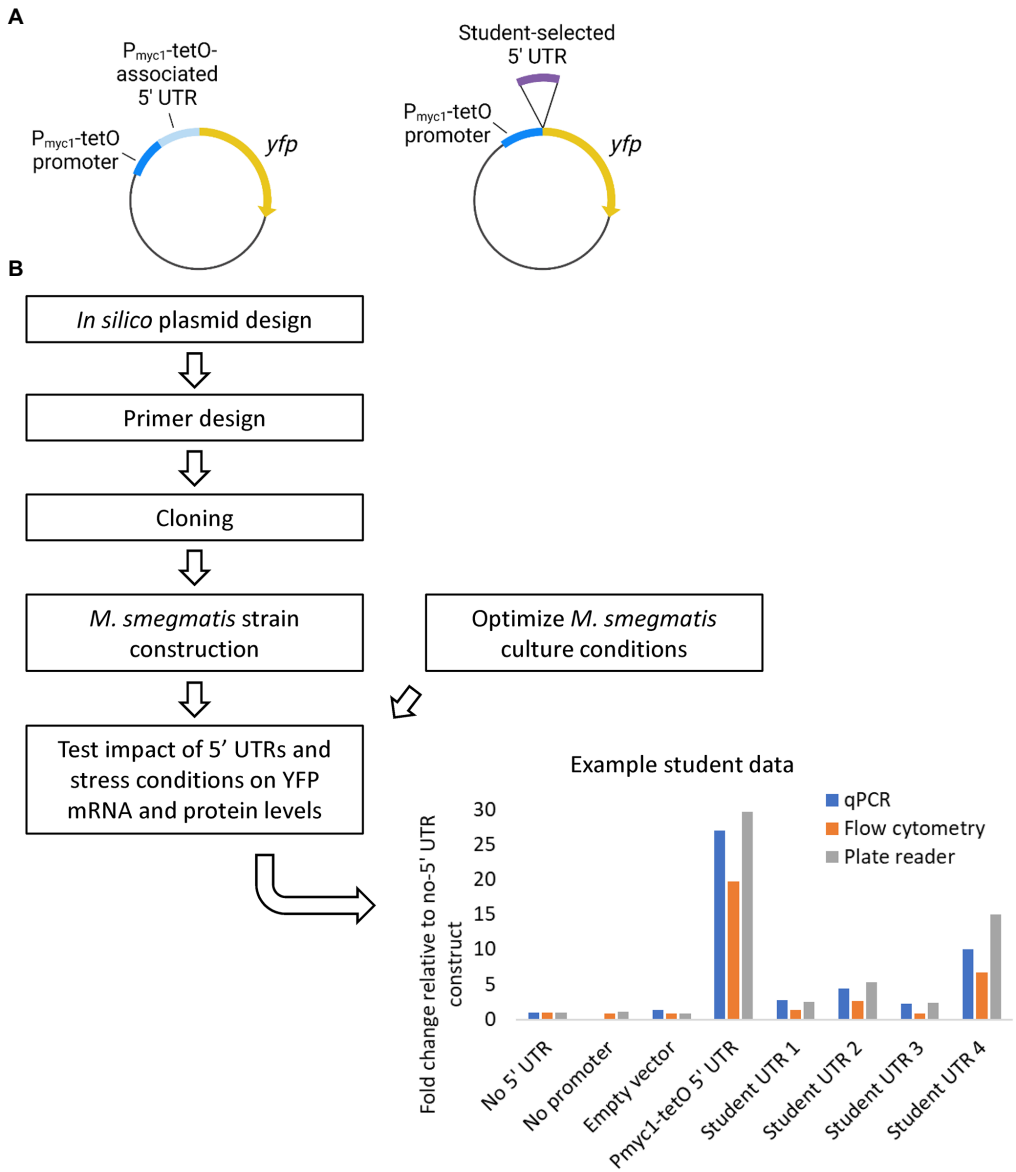
SCI learning gains, comapring in-person (*n* = 16 student responses) vs. entirely remote (*n* = 17) delivery for the MBGE lab.

We identified 16 specific lab skills (see Figure 3) we expected the students to master (learning objective 1). Of these, seven were largely *in silico*/electronic skills, and nine were defined as wet lab technical and procedural skills. Successful experimental outcomes and direct observation of students implied mastery. Students were required to design primers, determine the parameters for proper assembly, and design their experiments (learning objective 2). Successful completion of designs was determined prior to experimentation through discussions and lab notebook checks. Essentially all the *in silico* and wet lab procedures require quantitative or qualitative analyses (learning objective 3) upon completion. To address learning objective 4, designs, results, and analyses were presented informally during the lab, and more formally in a culminating poster session at the end of the term. Daily summaries and a final student reflection paper were used to assess written presentation skills. Students worked in defined, persistent small groups of two or three for the term, performing parallel procedures. These features of the course promoted and mandated coordinating with lab partner(s) and collaborating as a class (learning objective 5). We are relying on student comments solicited *via* university course evaluations and a “personal reflections” assignment to gage if students perceived they developed an understanding of the research process in life sciences research laboratories (learning objective 6). See Table 1 for a list of assessments mapped to learning outcomes.

Assessments were administered throughout and at the conclusion of the course (see Figure 2). The Midterm Course Feedback was done to assess how the students felt the lab was functioning while adjustments could still be made. Through this assessment students reported a high satisfaction level with the course after 3 weeks (particularly with the importance of their individual project to the research enterprise of Dr. Shell’s laboratory and the use of the integrated electronic lab organization software). Students did state the pace was very brisk and more discussion of concepts behind the procedures would be of benefit. To this end, a weekly “lab group meeting” was instituted where the students, instructor, and TA set aside an ~45 min block to discuss concepts.

### 3.2. The research projects

#### 3.2.1. Identifying research questions

To achieve our learning outcomes and maintain the authenticity of working at the frontiers of human knowledge, we chose research questions for MBGE that met the following four criteria. (1) The research tackled a question that had not yet been answered in published work or by unpublished experiments in the Shell lab. (2) The research topic was directly related to planned or ongoing work in the Shell lab. (3) The research question was best addressed by a series of experiments run in parallel using different strains, genes, or other features. For example, in the first research thrust below, students tested the effects of a set of 5’UTRs that we hypothesized to have regulatory functions. This was naturally conducive to multiple groups working in parallel because each group used similar methodologies to investigate a different 5’UTR. (4) The appropriate techniques for addressing the question involved molecular biology and genetic engineering. Over the six times MBGE has been offered, we addressed three distinct research thrusts, described below. We moved to new research thrusts when new questions became high priorities for the Shell lab or when a project started in MBGE was passed on to a full-time member of the Shell lab for more intensive investigation.

#### 3.2.2. Research thrust 1: Using reporters to evaluate 5’UTR roles

**Figure 4:**
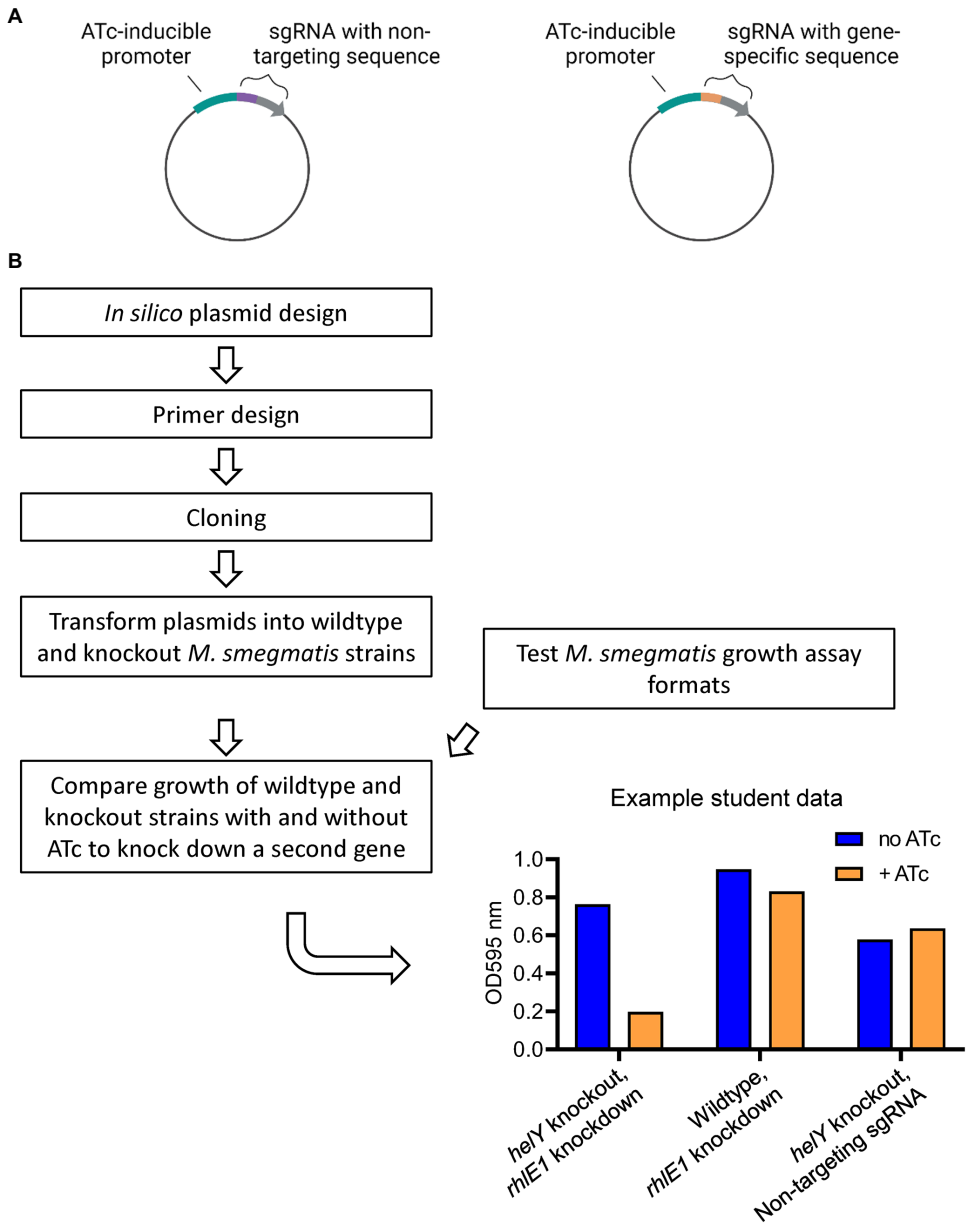
Research thrust 1: Using reporters to evaluate 5′ UTR roles. **(A)** Plasmids used to assess the effects of 5′ UTRs on expression of the reporter gene *yfp*. The P_myc1_-tetO promoter was used in the absence of a tet repressor to achieve constitutive high-level transcription. Plasmid elements not shown include and *E. coli* origin of replication, an antibiotic resistsnce marker, and sequences to integrate the plasmid into the *Mycolicibacterium smegmatis* chromosome by site-specific recombination. A plasmid with the P_myc1_-tetO associated 5′ UTR (left, pSS303) served as a positive control for efficient translation. Students used HiFi assembly (NEB) to insert *M. smegmatis* 5′ UTRs of intrest into a plasmid lacking a 5′ UTR (right, pSS310). Graphics created with BioRender.com. **(B)** Student workflow for plasmid construction, strain construction and expression testing. An example of data generated by students is shown.

For our first offering we focused on elucidating the potential roles 5’UTRs of transcripts may play in regulating gene expression at the mRNA and protein levels. We explored whether 5’UTR sequences alter the stability of a transcript or its translation into protein, using Yellow Fluorescent Protein (YFP) as a reporter. We custom-designed the plasmids and expression cassettes for this course. Each offering we altered and evolved these components; the most optimized versions are presented here in Figure 4. Our plasmid backbone contains the *E. coli* origin of replication; a hygromycin resistance gene for selection in both *E. coli* and *M. smegmatis*; and a sequence encoding a phage integrase enzyme along with the corresponding *attP* sequence for targeted integration. Our YFP reporter is expressed *via* the P_myc1_-tetO promoter (Ehrt et al., 2005), contains a C-terminal 6xHis tag, and is flanked by bi-directional transcriptional terminators (Nguyen et al., 2020) to isolate the expression cassette. Note there is no tet repressor in our system, so expression from P_myc1_-tetO is constitutive despite the presence of tet repressor binding sites. We have versions of this plasmid lacking any 5’UTR (pSS310), with a 5’UTR associated with the P_myc1_-tetO promoter that causes YFP to be expressed to high levels (pSS303), and a promoterless version (pSS314) to use as a negative control for expression assays (Nguyen et al., 2020).

The students designed primers to insert the 5’UTR of a gene likely to be regulated in response to stress (i.e., hypoxia, carbon starvation, or antibiotic treatment) into the plasmid. Students used Benchling to build the final, intended sequence of their plasmid with their 5’UTR inserted. Students used HiFi Assembly to insert their 5’UTR into pSS310 between the promoter and YFP, or “swap” with the P_myc1_-tetO -associated 5’UTR in pSS303. Upon transformation, selection, and PCR screening, the plasmids were miniprepped and sent for sequencing. Students aligned their sequences to the predicted and initial plasmid sequences in Benchling. Plasmids with the expected sequences were electroporated into *M. smegmatis*, transformants were selected, and integration was confirmed *via* PCR at both the left and right junctions using primer sets validated by the Shell lab.

While cloning was ongoing, students acquired mycobacterial culture skills and collected preliminary data they would need to design and execute effective experiments. For example, when studying 5’UTRs of ribosomal protein genes, students researched published minimum inhibitory concentrations (MICs) of ribosome-targeting antibiotics as a starting point to define sub-MIC concentrations that might reduce growth and induce stabilization of transcripts encoding ribosomal proteins.

Once cloning and strain creation were complete, the strains were then tested under these conditions to determine if the stress altered expression of YFP in a UTR-dependent manner. YFP protein levels were measured with a plate reader, by flow cytometry, or by western blot. *Yfp* mRNA levels relative to the housekeeping gene *sigA* were measured by qPCR using primer sets previously validated for qPCR by the Shell lab.

Primer design, cloning, and creating the *M. smegmatis* strains were completed in the first half of the course (by the 11th lab session). Consistently across the three offerings, ~75% of the groups successfully met the cloning objectives. All groups determined efficacious concentrations of antibiotics to use in their experiments. In the second half of the course students were able to chart cell growth under stress conditions and perform expression-level analyses. Representative data are shown in Figure 4, in which UTR-specific differences in expression are detectable. Note the relative abundances of mRNA (*via* qPCR) and protein (*via* fluorescence) correlate.

Subsequently, the plasmids created in the course were utilized in the Shell lab as a component of summer REU and senior research projects. One course alum who continued in the Shell lab and expanded upon their research from the course became a co-first author on a peer-reviewed publication in the Journal of Bacteriology (Nguyen et al., 2020).

#### 3.2.3. Research thrust 2: Using CRISPRi to assess functional redundancy in RNA degradation proteins

**Figure 5:**
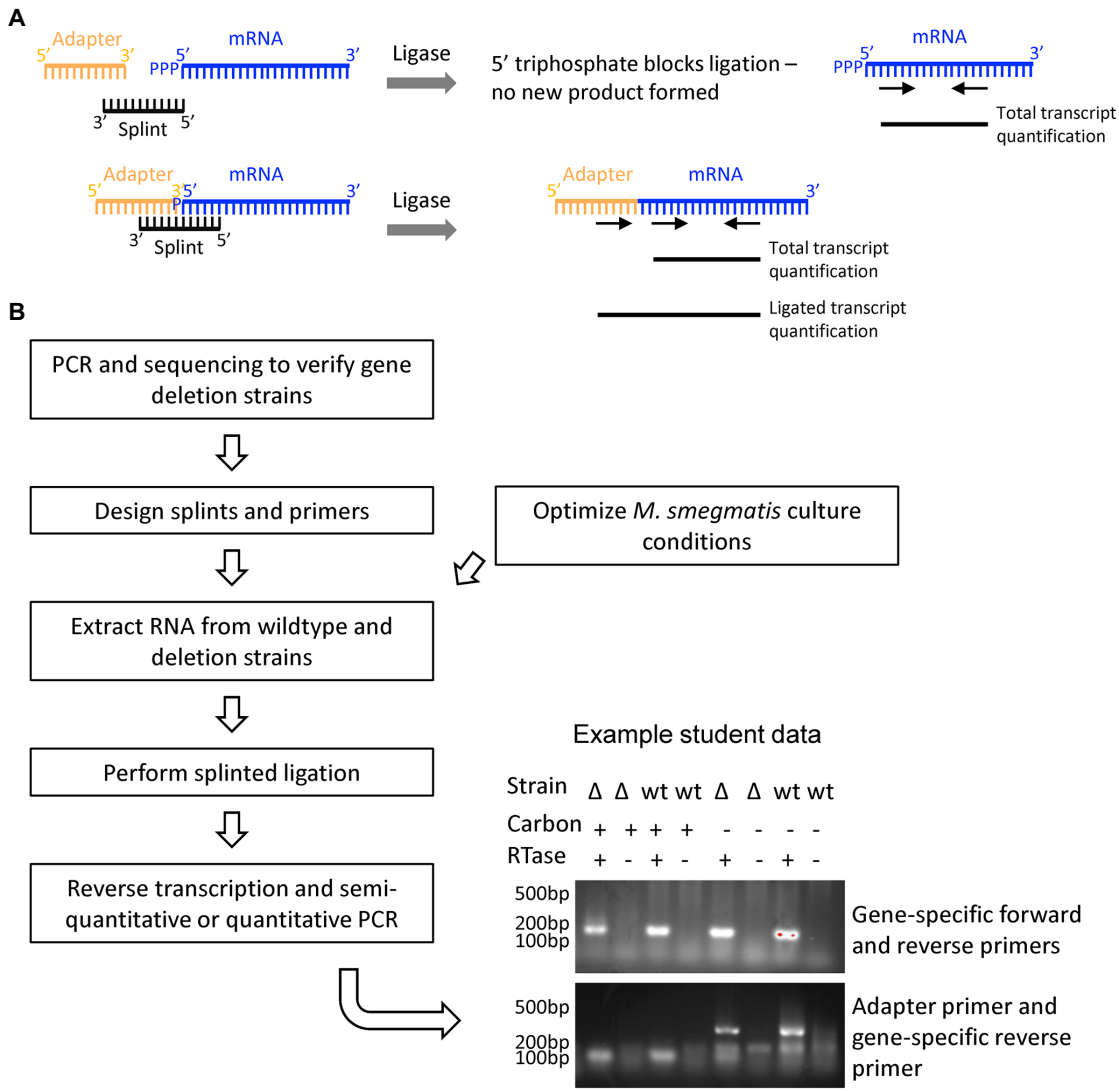
Research thrust 2: Using CRISPRi to access functional redundancy in RNA degradation proteins. **(A)** CRISPRi plasmids used to express catalytically dead Cas9 and sgRNAs. Students used HiFi assembly (NEB) to replace part of a non-targetting sgRNA with gene-specific sequence. Plasmid elements not shown include *dcas9*, an *E. coli* origin of replication, and anibiotic resistence marker, and sequence to integrate the plasmid into the *M. smegmatis* chromosome by site-specific recombination. Expression of the sgRNA and dCase9 was controlled by ATc-inducible promoters. Graphics created with BioRender.com. **(B)** Student workflow for plasmid construction, strain construction, and measuring growth. An example is shown of student data testing for functional redundancy between the RNA helicases helicases HeIY and RhIE1.

Identifying the roles of RNA degradation proteins in mycobacteria is a high priority because mutations in RNA degradation protein genes are associated with drug resistance in clinical *M. tuberculosis* strains (Hicks et al., 2018; Martini et al., 2022). Some essential functions in *M. smegmatis*, like RNA helicase activity, may be fulfilled by redundant genes that are individually classified as non-essential. Three putative RNA helicases exist in *M. smegmatis* (*helY*, *rhlE1*, and *rhlE2*) (Uson et al., 2015; Khemici and Linder, 2016; Hausmann et al., 2021), and none of these genes are essential. The Shell lab previously constructed deletion mutations for each of these three genes, and in each case, the knockout has no or a very modest growth defect phenotype. We hypothesized missing two of the putative helicase genes may result in reduced/no growth. To test this, we used an inducible CRISPR interference (CRISPRi) system (Rock et al., 2017) to knock down expression of one helicase in a knockout strain background where the gene encoding a different putative helicase is absent (Figure 5). Expression of *dCas9* and the single guide RNA (sgRNA) targeting the knockdown helicase are induced by ATc. We then may be able to detect ATc-specific reduction in growth by performing growth curve experiments in liquid culture.

Students designed sgRNAs to target their genes of interest, constructed CRISPRi plasmid sequences containing these sgRNAs *in silico*, and designed PCR primers to insert their sgRNA sequences into the CRISPRi plasmid. They then cloned the plasmids by HiFi Assembly (NEB), validated them by sequencing, transformed them into *M. smegmatis*, and verified correct plasmid integration into the *M. smegmatis* genome by PCR. While students were inserting their designed sgRNA into the CRISPRi plasmid, they determined what growth assay format would allow them to detect differences in growth rate of *M. smegmatis* cultures. Students compared growth in tubes (5 ml culture) vs. microplates (200 μl culture) aerated *via* shaking at 37°C, using sub-MIC concentrations of antibiotics to generate variable growth rates. Results from this preliminary test informed their experimental designs with the knockdown/knockout combination strains.

Finally, each student group compared growth rates for a series of strains and conditions: a wildtype strain with a non-targeting sgRNA with and without ATc; a wildtype strain with a specific sgRNA to knock down a gene of interest with and without ATc; a knockout strain with a non-targeting sgRNA with and without ATc; and a knockout strain with a specific sgRNA with and without ATc. We expect that if the genes knocked out and knocked down have partially redundant essential functions, there will be reduced growth of the knockout strain with the specific sgRNA in the presence of ATc.

Students were able to complete the experimental objectives of the course. Cloning and *M. smegmatis* strain construction were completed in the first half of the course, and three iterations of growth assays were conducted in the analysis phase. Students were able to identify strengths and limitations of the tube and microplate assay methods, and monitor growth rates of their strains relative to controls with and without ATc (Figure 5). Some growth reduction was observed for some knockout/knockdown combinations compared to the controls. While the cloning of sgRNA sequences into the CRISPRi plasmid was straightforward, teaching the complexities of the CRISPRi system required additional in-lab time as compared to Research Thrust 1. We attribute this to the fact that students are less familiar conceptually with CRISPR than with reporter genes, likely because CRISPR is a much more complex and recently emerging technique. This version of the course was offered for the first time in 2022; the next iteration will utilize a similar approach to investigate potentially redundant essential functions for a different set of genes. Logically, we would quantify the expression level of the knocked down gene *via* qPCR in order to confirm ATc-dependent repression; this was not performed in 2022 but will be incorporated in the next offering. The main outputs of the course for the Shell lab were clues toward which RNA degradation proteins may have redundant functions, and generation of 27 new *M. smegmatis* strains with a variety of knockout/knockdown combinations that are being used in undergraduate summer research experiences.

#### 3.2.4. Research thrust 3: Assessing mRNA 5′ end cap state *via* splinted ligation

**Figure 6:**
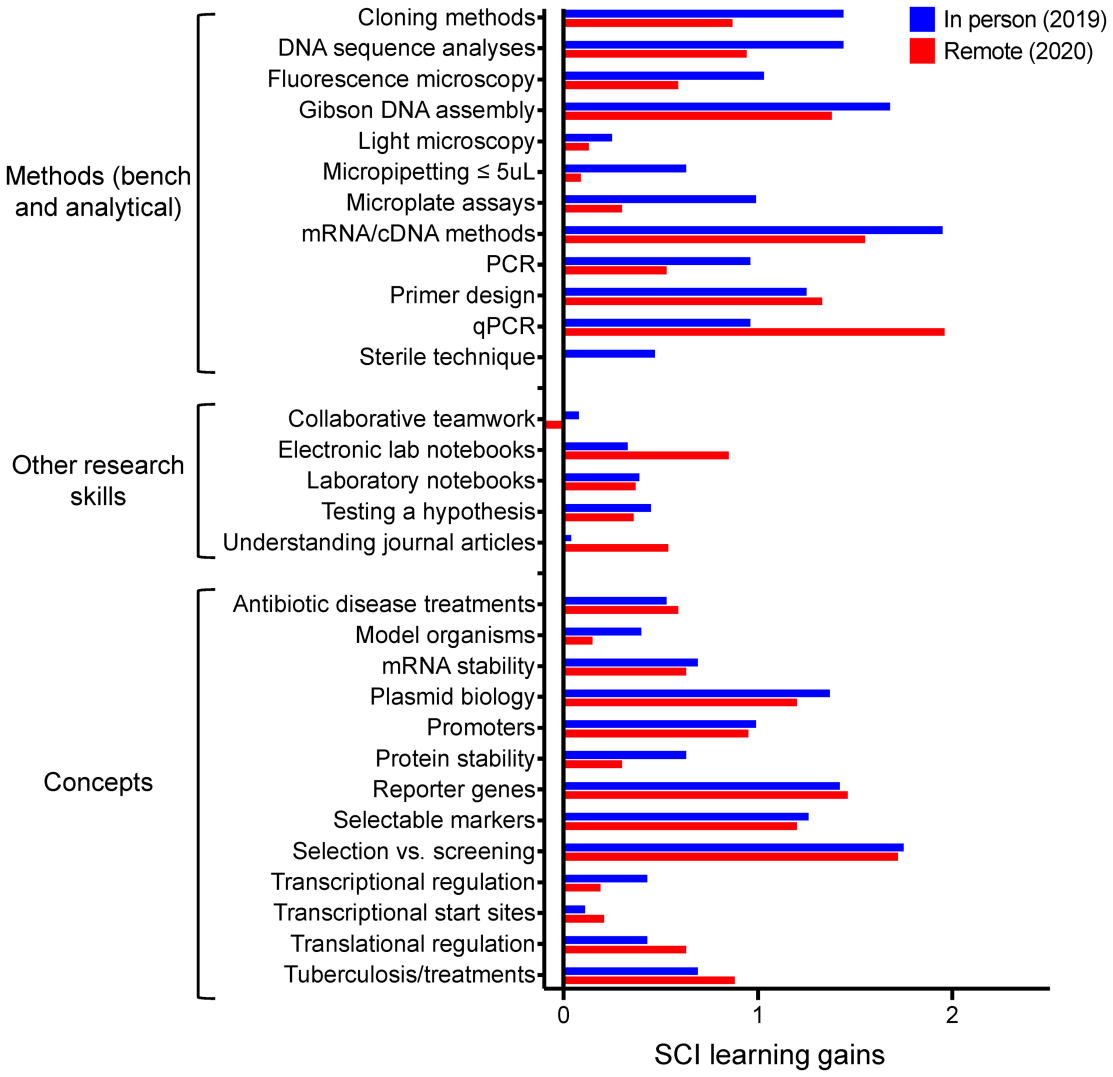
Research thrust 3: Assessing mRNA 5′ end cap state by splinted ligation. **(A)** The relative proportion of monophosphorylated mRNA 5′ ends was assessed by measuring the efficiency of ligation to an adaptor, which is dependent upon the presence of a 5′ monophosphate. mRNAs with 5′ triphosphates or other 5′ end chemistries are not ligatable. Primers that anneal internally within the transcript were used to quantify total transcript, and an adapter primer paired with and internal primer were used to quantify ligated transcript. **(B)** Student workflow for confirming the deletion of putative mRNA 5′-end acting genes in *M. smegmatis* strains obtained from a large collection (Judd et al., 2021), designing splints and primers to assess the 5′ end chemistry of putative target transcripts, optimizing standard and carbon starvation culture conditions, extracting RNA, and performing splinted ligation and PCR-based analysis of the ligation products. An example is shown of student data testing the impact of a gene deletion and carbon starvation on the ligatability of a target transcript.

The nature and importance of mRNA 5′ end chemistry is well-known in mammalian cell systems; less is understood about the relationship between 5′ end state and transcript stability in mycobacteria. Depending on how the mRNA is processed, the 5′ end may have a triphosphate, monophosphate, hydroxyl, or alternate (e.g., NAD) cap (reviewed in Vasilyev et al., 2019). Our research questions were (1) which enzymes are responsible for modifying mRNA 5′ end chemistry and (2) does the mRNA 5′ end chemistry change upon cell stress induced by carbon starvation? We focused on comparing the relative abundance of 5′ monophosphates, which are predicted to stimulate transcript degradation, compared to other 5′ end chemistries which are predicted to protect transcripts from degradation. We hypothesized that one or more of a set of candidate proteins was responsible for converting other 5′ chemistries to 5′ monophosphates, and that a larger proportion of transcripts would exist in the triphosphorylated state in carbon starvation conditions where mRNA degradation is known to be slowed. Our methodology is based on the fact that only 5′ monophosphorylated mRNA species can be ligated to an adapter, and therefore by comparing the relative proportion of ligatable 5′ ends in different strains and conditions we can infer the relative proportion of 5′ monophosphorylated transcripts (Figure 6).

Students first used PCR and sequencing to verify a set of *M. smegmatis* strains with deletions of genes predicted to act on RNA 5′ ends (Judd et al., 2021). They then designed qPCR primers and splints to increase the efficiency of ligation of an adapter to specific transcripts. Aware of cost, we chose to ligate a 50 nt single-stranded DNA adapter to 5′ monophosphorylated transcripts using T4 DNA ligase (high concentration; NEB M0202M) rather than an RNA adapter as was used in published protocols (Celesnik et al., 2008; Blewett et al., 2011). Total transcript levels were quantified *via* qPCR using primers designed to bind within the transcript itself. Relative 5′ monophosphorylation levels were quantified by qPCR using a forward primer that binds the adapter in conjunction with the reverse internal primer. We could then qualitatively determine the relative proportion of 5′ monophosphorylated transcript across strains and conditions.

Students grew cultures of wildtype and deletion strains both in log phase and carbon-starvation, extracted RNA, carried out the splinted ligation procedure, synthesized cDNA, and finally used semi-quantitative PCR to assess relative 5′ end status.

This offering had a focus on methods to isolate, modify, and measure RNA. While primarily motivated by initiatives in the Shell lab, this version was also inspired by the pandemic-induced awareness of RNA methods and technologies. This tangible connection to the real world was easily perceived by the students, and represented a unique advantage in training students to think about and handle RNA. The techniques of RNA manipulation require a high level of skill, focus, and precision; experimental success was more limited due to the relatively low level of experience of nascent student scientists. Though unintentional, the limitations in obtaining RNA reagents exposed students to alternative methods of cDNA synthesis (transcript-specific primers were used at first because random hexamer primers were temporarily unavailable). In addition to providing a valuable skill set to the students, a significant outcome of the course was protocol development. Our splinted ligation protocol was based upon two salient methods papers (Celesnik et al., 2008; Blewett et al., 2011) and modified to work well in our system. The students were able to verify that the protocol worked, and potentially observe some condition-specific differences in 5′ end chemistry (Figure 6). A postdoctoral researcher in the Shell lab is now using this methodology to investigate 5’ RNA end status in *M. tuberculosis*.

### 3.3. Assessments of course and student learning

#### 3.3.1. Course-based undergraduate research experience, LCAS, and university course evaluation assessment data

We wanted to use national, standard assessments to compare our existing, traditional skills-based labs with the new authentic research upper-level course. Notably, the MBGE lab was the first full-credit lab offered by our department, based upon the research program of one of our tenure-track faculty, and filling a hole in our lab curriculum. Thus, we conducted a broad range of assessments to determine how well this course met the objectives. We utilize the Course-based Undergraduate Research Experience (CURE) assessment tool (CURE survey, 2022; Auchincloss et al., 2014) administered at the beginning and end of the term, with a particular focus on the end-of-term student perceptions of their learning and understanding of general scientific principles. We have previously reported on the CURE end-of-term assessment data [Buckholt et al., 2022 (in press)] for this course, in comparison to a skills-based molecular biology lab we offered. The CURE survey data show the students reported larger gains in broad and specific skills, with 91% of students emerging from MBGE with increased self-efficacy and confidence as scientists.

The Laboratory Course Assessment Survey (LCAS) (Corwin et al., 2015) was also used for this new course, specifically to assess the discovery/research course outcomes upon completion of the lab; these data are reported in Table 3. The LCAS and CURE survey results reflect this intensive research-based lab course successfully met its learning outcomes for the students, who were able to identify that they created new scientific knowledge by generating novel results answering their research questions. Taken together, these assessment results show the students enrolled in MBGE felt more invested in the experiments, gained a clear understanding of how theory and knowledge are integrated into design of their projects, and emerged confident to face the challenges of original scientific research in the future.

**Table 3:** LCAS survey discovery/research results.

In this course I was expected to…	SD	D	~D	~A	A	SA
Generate novel results that are unknown to instructor and that could be of interest to the broader scientific community or others outside of the class	1	1	0	1	11	9
Conduct an investigation to find something previously unknown to myself, other students, and the instructor	0	0	2	4	12	5
Formulate my own research questions or hypothesis to guide an investigation	0	0	0	5	8	10
Develop new arguments based on data	0	0	1	6	9	7
Explain how my work has resulted in new scientific knowledge	0	0	0	3	9	11

*n* = 23 students, in-person instruction in 2017 and 2021. SD = strongly disagree; D = disagree; ~D = somewhat disagree; ~A = somewhat agree; A = agree; SA = strongly agree; no students responded “I do not know” or “I prefer not to respond.”

WPI deploys course evaluations completed by the students in the final class week to assess the structure, delivery, and outcomes of the course, as well as the teaching effectiveness of the instructor WPI course evaluations are used in all classes, which allows comparisons within our department curriculum, but are not specifically designed for lab courses. Relevant WPI student course evaluations data are presented in Table 4, using a skills-based “cookbook” predecessor course as a comparator. Course quality, educational value of the assigned work, and the amount learned scored much higher for the MBGE lab as compared to the skills-based version. In 2020 the MBGE course was offered fully remote due to the pandemic. Interestingly and perhaps reassuringly, much of the value in performing authentic research was lost in the remote environment when data sets from past versions were utilized in place of hands-on exploratory research.

**Table 4:** University student course report data.

Question text	2016^#^ (*n* = 10)	2017 (*n* = 11)	2018 (*n* = 15)	2019 (*n* = 8)	2020* (*n* = 15)	2021 (*n* = 8)	2022 (*n* = 11)
My overall rating of the quality of this course is	4.0	4.7	4.8	4.3	4.1	5	4.9
The educational value of the assigned work was	4.3	4.6	4.7	4.1	4.4	5	4.8
The instructor’s organization of the course was	4.2	4.3	4.6	3.9	3.9	4.9	4.4
Relative to other college courses I have taken, the amount I learned from the course was	3.6	4.5	4.6	4.5	4.3	4.8	4.8
The instructor stimulated interest in the subject matter	3.5	4.6	4.7	4	4.7	4.6	4.9
Relative to other lab experiences, the intellectual challenge presented by the lab assignments was	4.0	4.2	4.2	3.9	4.3	4.4	4.4
Relative to other lab experiences, the clarity and specificity of lab assignment objectives was	3.3	4.1	4.4	4.1	3.9	4.8	4.5

*n* = number of student responses. *Offering was fully remote due to pandemic. ^#^“2016” is a comparator skills-based molecular biology lab replaced by the MBGE rpl-CURE.

#### 3.3.2. Skills and concepts inventory assessment

The MBGE course was created to immerse upper-level undergraduates in authentic research projects, while maintaining the skills development offered in more recipe-based labs. While the CURE and LCAS surveys do a good job at ascertaining student perceptions about how their skills have developed within the course, the questions are posed generally rather than about specific techniques. It is important to us that the students also understand the concepts that underpin these skills, in order to understand their applicability, strengths, and limitations. The LCAS is only administered upon conclusion of the course, and thus cannot reliably or quantitatively assess learning gains. Thus, we set out to devise a novel assessment tool we call the Skills and Concepts Inventory (SCI) to assess learning gains focused on the specific skills and concepts we are teaching in this course (Document S1 and Roberts and Shell, 2022).

The SCI is administered on the first and last lab day; both versions are identical except for the wording of the prompt. Students are asked to rate their familiarity/comfort/expertise on a 0–4 scale; 0 represents no familiarity, while 4 indicates a student believes they have expertise in that skill or concept. By comparing student responses at the beginning and end of the course, learning gain can be quantitatively assessed (learning gain = final – initial). A learning gain of zero indicates no gain of knowledge. Gains are relative to perceived incoming knowledge, thus negative learning gains are possible. One limitation of the SCI is the reliance on student self-assessment (which is also true of the CURE survey, LCAS survey, and course evaluations). An additional limitation is that absolute learning gains are greatly affected by initial rating of a skill or concept by the students; if many students enter with a high degree of familiarity/comfort/expertise, there is less of a range of learning gain possible. Thus, normalized learning gains are utilized to account for variations in initial assessment scores (Coletta and Steinert, 2020).

The SCI allows us to probe how well the course improved student understanding at a high-resolution level of individual skills and concepts, as opposed to more broadly at the course objectives level. For MBGE we identified 30 skills and concepts we hoped the students would learn by completing this course, from general techniques (e.g., micropipetting, electronic lab notebooks, etc.,) through class-specific methods (e.g., mycobacterial cell culture, fluorescence quantitation, qPCR, etc.,). We quantified both raw and normalized learning gains for skills and concepts grouped into three categories- methods, other research skills, and concepts. Our results from two offerings are presented in Figure 3 and Supplementary Table S2. Positive learning gains were reported by the students for 29 of the 30 skills and concepts, even when the course was offered remotely. Generally, the in-person offerings report more skills development as compared to the remote version. Concepts emphasized when the lab was remote such as understanding journal articles and how qPCR data are obtained and analyzed reported higher raw and normalized learning gains than those obtained from in-person instruction. Inspired by this result, we have recently created a qPCR learning module the students can access *via* our learning management system (Canvas).

#### 3.3.3. Additional course-specific assessments

When a lab course is run for the first time, we often utilize a midterm assessment to determine if the class is tracking along as intended, or requires in-term modification. The Midterm Course Feedback questionnaire, adapted from one created by the Committee on Academic Issues of the Student Government Association at WPI, includes items rated on a 1–5 scale, along with open-ended questions particularly relevant to students’ interests and concerns. This survey was deployed at the start of the 4th week of the initial offering. For further offerings we substituted a discussion board-formatted “Three Questions to Address” and “Most Interesting/Least Clear” discussion board postings. Additionally, an “instructor reflections” document was prepared upon conclusion of each course offering. We used data from all surveys to assess, modify, and evolve the lab course.

#### 3.3.4. Toward assessing student exposure to how academic research is done

A motivation for this course is to truly demonstrate how extramurally funded academic research is conducted. Specifically, we wanted the students to experience the “purposeful fluidity” of the research process- how research directions are chosen and evolve over time. We sought to construct a course that would convey to the students how specific problems/questions/gaps in knowledge are identified, and how research scientists design experiments to provide data to address those gaps. In practice, assessing if students met this learning outcome is a challenge. First, most course assessments are administered within the confines of the term, and not longitudinally for individual classes. We feel standard, quantitative assessments are unable to put learning into a future context, which is what this learning outcome would require. However, we feel student comments solicited on university course evaluations and personal reflections essays can provide insight into whether students felt exposed to the academic research process. We also wanted to determine if the students saw value in the involvement of the research-active faculty member (and lab) as collaborators.

University course evaluations prompt students to express what they liked/disliked about each class, and whether they would recommend the course to other students. Representative sample comments include:

“I liked that this lab involved a real research experience. It was designed to follow a general series of steps but each group would have had individual differences based on their previous results.”- 2019.

“This class is what education should be: a true synthesis of theory and practice, tailored to each students’ interests while actively contributing to ongoing and critical, life-saving research.”– 2021.

“I really liked what we were researching and that is was a new road for everyone including the professor so it was fun to have to change as we went along.”- 2021.

“Feeling like I was doing authentic research was something that motivated me to get out of bed every morning genuinely excited to see what we were doing in lab that day.”- 2022.

At the conclusion of each offering, students wrote a “personal reflections” essay summarizing their research finding, and their experience in the class. Though neither quantitative nor exhaustive, many students expressed an appreciation for, and perceived their role and value in, the research process. Representative comments include:

“Being able to guide experiments while having class discussions of our findings and next steps was incredibly valuable to my growth as a researcher.”- 2022.

“I am confident in saying that this course assisted my acceptance to graduate school. It equipped me with real skills that I will continue to utilize in the next 4 years, but it also instilled a greater confidence that I do know some things and what I do not know I can always learn.”- 2021.

We were encouraged that the authenticity and value of the research process was palpable to the students who completed this course. We find it critically important and satisfying that students began to see themselves as contributing scientists, where this laboratory had a positive impact on their career trajectories.

## 4. Conclusions and future directions

Our goal was to thoughtfully create a new laboratory course that would engage students in truly authentic, extramurally-funded research. We devised a framework to build our rpl-CURE, and applied it to create a lab experience in which students could use the modern methods of molecular biology to generate novel results of value beyond the course itself. The MBGE lab also serves as a model to further evolve our lab curriculum toward discovery-based science. We assessed the effectiveness of our approach using standard (broad) and novel (specific) tools, with a focus on meeting the learning outcomes we identified, and fostering a means to allow students to identify themselves as self-efficacious scientists.

MBGE ran for the first time in 2017, and our assessment data showed students successfully met all six stated learning outcomes by the conclusion of the course. Each year we reassessed the focus of the course and chose research questions that honor the exploratory nature of research. These research questions were formulated as directed by ongoing research in the Shell lab, and our delivery was guided based on what we learned from previous offerings and assessments of the course. To date we have utilized three unique research thrusts, all of which have fulfilled our goals of meeting learning outcomes, conducting authentic research, and supporting student confidence in their ability to conduct scientific inquiry. The projects undertaken retained authenticity, had longevity, and provided preliminary data and/or methods later used for sponsored summer research, independent studies, senior capstone research, graduate student rotation and thesis projects, and postdoctoral projects. Notably, a student course alum is a co-first author on a peer-reviewed research publication from the Shell lab that focuses on the roles of 5’UTRs in mycobacterial gene expression regulation (Nguyen et al., 2020). In its first offering in 2022, the CRISPRi-based thrust has generated a series of sgRNA-expressing plasmids and *M. smegmatis* strains that will be utilized in both the Shell lab and for future iterations of this thrust within the course. The main output from the RNA-focused version was developing a splinted ligation procedure to fuse DNA adapters to specific transcripts. Taken together, the results, materials, and protocols generated within the MBGE lab course are of wide scientific value and utility.

The free web-based platform Benchling to import, create, edit, annotate, and analyze DNA sequences was well-suited for an upper-level molecular biology lab course. The students and teaching staff also found the notebooking feature in Benchling intuitive and appropriate. We settled on each pair of students creating one Benchling project and joint notebook. This approach was consistent with the collaborative nature of the research, harbored the portability of sequences to share with the class and archive for the Shell lab, and accommodated lab notebook grading.

The unique circumstances imposed by the pandemic allowed us to separate wet lab hands-on skills from concepts, providing unique insight into how students assimilate and utilize information provided by completing the course. Data indicate that concepts that were focused on remotely promoted student learning to a comparable level to in-person instruction. Unsurprisingly, skills development was shown to be reduced by operating in a solely remote environment. We view the alignment between our data and the logic that skills are best taught in a hands-on fashion as evidence of the validity of the SCI assessment tool. Recently we converted the SCI into a Qualtrics survey format to better extract the data and broaden its utility, and have deployed it in additional undergraduate and graduate lab courses. With regards to assessments, we recommend the student reflections prompt more explicitly guides students to provide responses that can be coded within a qualitative data analysis framework. This may better reveal to what degree the students self-assessed as gaining an understanding of the research process, and viewing the collaborative nature of the scientific work as valuable. The student reflections thus can be leveraged for providing an evaluation of the course, in addition to their original purpose of assessing how well a student could articulate their research project objectives, workflow, and results.

MBGE has been offered six times, with three different research thrusts based on one research active faculty member. We can envision the research enterprises of other faculty members in our department serving as inspiration for the course (e.g., cloning and testing metal biosensors in bacteria). Beyond molecular biology approaches and applications, we used a similar framework to create a “Cell Culture Models for Tissue Regeneration” laboratory course. We designed this course for up to 20 upper-level students per offering. With regards to scalability, the main considerations are more resource-limited (instructional capacity and cost) rather than constrained by experimental space to explore. We do feel the fluid nature of the active research process requires the direct instruction by a PhD level scientist/faculty member. We believe the strategy we present here is widely applicable to create upper-level authentic research experiences to effectively develop and inspire a greater number of student scientists than research labs alone can accommodate.

## Data availability statement

The original contributions presented in the study are included in the article/Supplementary material, further inquiries can be directed to the corresponding author.

## Author contributions

SS conceived the research projects. LR and SS designed the experiments and wrote the manuscript. LR designed the course logistics, selected and administered assessments, and taught the course. All authors contributed to the article and approved the submitted version.

## Funding

This work was supported in part by NSF CAREER award 1652756 to SS.

## Conflict of interest

The authors declare that the research was conducted in the absence of any commercial or financial relationships that could be construed as a potential conflict of interest.

## Publisher’s note

All claims expressed in this article are solely those of the authors and do not necessarily represent those of their affiliated organizations, or those of the publisher, the editors and the reviewers. Any product that may be evaluated in this article, or claim that may be made by its manufacturer, is not guaranteed or endorsed by the publisher.
